# Hormone-Balancing Effect of Pre-Gelatinized Organic Maca (*Lepidium peruvianum* Chacon): (I) Biochemical and Pharmacodynamic Study on Maca using Clinical Laboratory Model on Ovariectomized Rats

**Published:** 2006-09

**Authors:** H. O. Meissner, P. Mrozikiewicz, T. Bobkiewicz-Kozlowska, A. Mscisz, B. Kedzia, A. Lowicka, H. Reich-Bilinska, W. Kapczynski, I. Barchia

**Affiliations:** 1 *Faculty of Health Studies, Charles Sturt University & Therapeutic Research International, GPO Box 4792, Sydney 2001, Australia;*; 2 *Research Institute of Medicinal Plants, 27 Libelta St., 61-707 Poznan, Poland;*; 3 *Department of Pharmacology, Medical University, Poznan, Poland;*; 4 *Specialist Gynecology Private Clinic, Glogow, Poland;*; 5 *Specialist Gynecology Private Clinic, Poznan, Poland;*; 6 *Department of Primary Industry, E. Macarthur Institute, Menangle, Australia*

**Keywords:** blood hormones, gelatinized Maca, ovariectomized rats, pharmacodynamics, toxicity

## Abstract

Ovariectomized rats were used in a model laboratory study to examine biochemical and pharmacodynamic effects of pre-gelatinized organic preparation of *Lepidium peruvianum* Chacon (Maca-GO). Biochemical and Pharmacodynamic effects of Maca-GO (250 mg Maca-GO per kg body weight (bw) administered by intubation twice daily) were assessed in a 28 day model laboratory study on ovariectomized (by laparoscopy) Wistar rats with pharmacodynamic tests performed at the conclusion of the trial followed by blood collection for morphology and biochemical tests. Toxicity of Maca-GO used in the study was determined in bioassay on mice and rats. Anti-depressive function (Porsolt’s test) and anxiolytic sedative and cognitive effects (using elevated-plus maze, locomotor activity and passive avoidance tests) were assessed against control (laparotomized female rats with intact ovaries). In addition to blood morphology, the following blood serum constituents were analyzed: Estrogen (E2), Progesterone (PGS), Cortisol (CT), Adrenocorticotropic Hormone (ACTH), Thyroid Hormones (TSH, T3, and T4), Iron (Fe) and lipid profile (Triglycerides, Total Cholesterol, LDL, HDL). Analytically-determined non-toxic status of Maca-GO was confirmed in bioassays when applied to mice and rats at levels of 0.5 and up to 15mg/kg bw which shows it safe use in humans with the LD_50_>15 mg/kg bw. Maca-GO showed a distinctive, (*P*<0.05) antidepressant-like and sedative effect in ovariectomized rats only, while there was no anxiolytic activity nor disturbance of cognitive function observed in both, test and control animals. Observed in this study balancing effect of Maca-GO on sex hormone levels show its potential as a safe preparation for use in correcting physiological symptoms characteristic in postmenopausal stage with an indication of potentially even more value for its use in pre-menopausal women.

## INTRODUCTION

Roots of Maca (*Lepidium peruvianum*) described by Chacon (1) and Obregon (2) are widely used as a dietary supplement in Peru and processed Maca preparations, namely Maca Root and/or Gelatinized Maca in powder, tablet or capsule form, are exported for international distribution by numerous trading and nutriceutic companies under generic name “Maca” and/or under various commercial trade mark names. Botanical and genetic characteristics and various biochemical and therapeutic properties of the root have been extensively reported (3-12).

Since the first report of Chacon on medicinal properties of Maca root (3), it has been generally accepted and confirmed in number of studies, that this plant doesn’t contain plant estrogens or any other phyto-hormones (13-17). It is considered that through plant sterols, Maca stimulates endocrine system helping to maintain hormonal balance (1) in a way that is not yet well understood (2, 18). According to Muller (6), these sterols are used by the body with the help of the pituitary to improve adrenal function, ovarian and testicular function, as well as the functioning of the thyroid, the pancreas and the pineal gland. Multi-functional effect of Maca on endocrine relationships may also explain reported in the literature, its positive influence on stimulation of endocrine glands in regulation of hormonal balances in the body (4, 10) and particularly in women entering a perimenopausal state of life.

From well known herbal alternatives widely used already as substitutes for synthetic hormones in the hormone replacement therapy (HRT) (4, 6), standardized extracts from soy, red clover, black cohosh, wild yam or licorice root are the most commonly applied in practice with well scientifically documented reviews accepted throughout (phyto-) pharmaceutical industries and medical profession (4). However, unlike the phyto-estrogenic herbs mentioned above or HRT, which affect one (black cohosh-LH only, wild yam – Progesterone only, red clover and soy – Estrogen only) or two hormones (HRT – Estrogen and Progesterone), Maca root works in an entirely different way for most women (1, 5), by promoting optimal functioning of the hypothalamus and the pituitary, thereby improving the functioning of all the endocrine glands. Original research conducted by Chacon (3) revealed that, alkaloids in the Maca root, produced fertility effects on the ovaries and testes of rats. She has further deducted that the alkaloids were acting on the hypothalamus-pituitary axis, which may explain why the effects in humans are not limited to ovaries and testes, but also acts on the adrenals, giving a feeling of greater energy and vitality and on the pancreas and thyroid as well.

Currently, standardized plant preparations from red clover or soy are most commonly used in helping treat menopausal symptoms. Their action is based on flavonoids with steroid molecular structure, resembling human hormones (19). They act reasonably fast but some of their side effects may be similar to synthetic hormones. On the other hand, alkaloids like those present in Maca are non-steroid compounds (1, 3), which are characterized by better tolerance by women who use it (6).

In previous papers from this series (11, 12, 20) on Gelatinized Organic Maca root preparation (Maca-GO), its positive physiological effect in both, post- (11) and perimenopausal (20) women was demonstrated, as well as results from short- and long-term bioassays on laboratory animals (sexually-active adult female and male rats) were presented (12). The observed effects on animals were gender and dose dependent, with different responses observed during a short- and a long-term Maca-GO administration (12). In addition to, appropriate to the gender, balancing effects of Maca-GO on hormones FSH, E2 and progesterone, positive results were also recorded in terms of tendency to restrict weight increase, lowering triglycerides in blood plasma and an increase in calcium and phosphorus deposition in bone and muscle tissues. The obtained results suggested that Maca-GO may be of value as a potential substitute to HRT in alleviating perimenopausal symptoms, or reducing dependence on HRT programs. Laboratory assays on male and female rats demonstrated also that Maca-GO may also be considered as a non-hormonal energizing supplement, assisting not only physically-active people and sportsmen, but in those women complaining on lack of energy and stamina, often experienced by women entering menopausal stage of life (12).

The above study confirmed functionality of Maca as herbal remedy used for centuries by natives of Peru in helping to treat conditions affecting menopausal women, although, the role of this plant product in releasing of steroids and/or affecting the hypothalamo-pituitary-ovarian axis is far from being well understood. Amongst wide spectrum of traditional uses, Maca is most frequently applied for energy, hormone balancing, healthy thyroid functioning, sexual functioning, pre-menstrual syndrome (PMS), menopause, as well as to help maintain healthy bones, as a tonic for elderly and assisting in convalescence. Maca has also been mentioned as helping women to alleviate variety of unwanted psychological experiences and stress-related symptoms - amongst them most predominant being emotional symptoms such as depression and frustration (20), commonly experienced during perimenopause and early postmenopause. However, most of broad, traditionally-claimed spectra of biological activity and functionality of Maca are not yet well documented nor experimentally proven (6).

Since anti-depressive and anti-stress function of Maca has not been experimentally demonstrated in relation to responses of menopausal women under clinical regimen conditions, therefore, in this study, an attempt has been made, firstly (part I of the paper), to determine anti-depressive and anxiolytic effect of pre-gelatinized organic Maca root powder in a clinical model on groups of laboratory animals with surgically-removed ovaries and with intact ovaries, and secondly (part II of the paper), to relate these results to a clinical observations made in a double blind, randomized, placebo-corrected multi-centre clinical study on early-postmenopausal women.

In this study the following aspects of Maca were investigated:

- Determining acute toxicity of Maca-GO after single oral application (by intubations) in mice and rats.
- Pharmacodynamic assessment of chronic application of Maca-GO to laboratory animals with surgically-induced ovarian dysfunction: 1. The effect of Maca-GO on hormonal activity along the axis: (a) Hypothalamus - pituitary – ovaries (FSH, LH, estradiol, progesterone). (b) Hypothalamus - pituitary- adrenal system (ACTH, Cortisol). (c) Hypothalamus – pituitary – thyroid gland (TSH, T3, T4). 2. The effect of Maca-GO on blood morphology and blood plasma iron and lipids levels. 3. The effect of Maca-GO on behavioral and cognitive functions: (a) anti-depressive action. (b) anti-anxiety action. (c) motoric action. (d) assessment of a long-term cognitive function.

## MATERIALS AND METHODS

### Maca

The plant species used in this study has been described in monographs by Chacon (1) and Obregon (2) as well as in the previous publications from this laboratory (11, 12).

Roots of the plant cultivated in Junin area (Central Andean Region of Peru between 4200 m and 4500 m altitude), were harvested and dried on plantation site and represented typical distribution of three main (out of 13 known) ecotypes predominant in this cultivation area: black, yellow and purple/red roots. Sun-dried Maca roots were transported and processed (gelatinization by a proprietary extrusion process without chemical treatment) in a processing plant at the National Institute of Agricultural Research (NIAR), National Agricultural University La Molina in Lima (Peru) after previous attestation of its organic status (formal certification by SKAL International Certification, Control Union World Group, as “organic”) and its independent authentication as cultivated Maca - *Lepidium peruvianum* Chacon.

Degree of gelatinization in the final powdered product (Maca-GO) was 99.8%. The processing of Maca was identical to the one described in the previous papers from this series (11, 12). The composition of the pre-gelatinized Maca-GO powder (batch TTD-ZMP-20100351) used in Part I and II of this study was reported previously (11). Maca-GO was then used in making hard gel vegetable capsules (500mg per capsule) for use in clinical study on early postmenopausal women (Part II of the paper). Identically-looking Placebo capsules contained sorbitol & cellulose powder only.

### Laboratory animals

- Male and female Swiss mice weighing between 18 g to 22 g were used in toxicity study (A).
- Male and female Wistar rats weighing between 180 g to 220 g were used in toxicity study (A).
- Sexually-experienced female Wistar rats weighing between 330 g to 370 g were selected for laparoscopy procedure prior to use in behavioral study (B).

Animals were kept in plastic cages (60 × 40 × 40 cm), 6-8 animals per cage in a laboratory under standard environmental conditions maintained at average temperature 20°C, 60% relative humidity and 12 hours light and dark cycle. They were fed standard pelletized diet (Labofeed B) and water available *ad libitum*.

All study on laboratory animals were conducted in compliance with relevant European standards and according to principles of animal protection as per relevant regulations enforced by Polish Law (Dz. Ust. Nr 111, poz.724 dated 23.09.1997) and approved by the Local Bioethics Committee at the Pharmacology Faculty, Medical University in Poznan, Poland – 35/2003.

### Acute Toxicity of oral doses of Maca-GO

The OECD protocol (22) was used adopting procedure to study acute toxicity of chemical substances. Mice were allocated into two groups, each of 5 males and 5 females. After starving for 24 hours, animals were force-fed with 2 ml water suspension of Maca-GO as 2 g/kg body weight (BW) by intubation (using stainless steel tube).

During the 14 day testing period, the following observations were made: any changes in skin, fur, eyes, mucosal membrane, abnormal respiratory rhythm, any abnormalities in blood circulation and nervous reactions in terms of motoric functions and behavior, with particular emphasis on such symptoms as shivering, convulsions, excessive salivation, diarrhea, lethargy, sleep and sleepiness/coma.

Similar study, in identical configuration as in mice were conducted on male and female rats with the dose of 2 g Maca-GO per kg BW (15 ml suspension in distilled water).

On conclusion of the trial all animals were weighed, put into sleep using CO_2_ and were subjected to macroscopic assessment of the body and organs.

### Measurements

**Mice:** Male mice allocated into four groups of eight animals each, were administered Maca-GO as 2 ml distilled water suspension by intubation in increments 1.0; 5.0; 10 and 15 g/kg BW.

**Rats:** Male rats allocated into four groups of eight animals each, were administered Maca-GO as 15 ml distilled water suspension by intubation in increments 0.5; 1.0; 2.0 and 5 g/kg BW.

After administration of the appropriate dose, animals were observed for the following 72 hours in terms of: (a) behavior (breathing rhythm, excessive salivation, convulsions, muscle spasms, mobility etc). (b) appearance (skin, fur, eyes, mucosal membrane).

The LD_50_ dose was determined on the basis of 50% animals died in each group, according to Litfield and Wilcoxon, applying computer model by Tallarid and Murray as per OECD Directive 408 on conducting toxicity study (22). However this program used in assessment of pharmaceutical trials could not be applied in the present study since there were no death recorded in any of the groups at the doses of Maca-GO used, which would justify use of the computerized calculations. Applying higher doses of Maca-GO was impractical due to maximal volumes possible for single intubation: 5 ml per mice and 15 ml per rat.

### Ovariectomy procedure

Sexually-experienced 72 Wistar female rats were selected to the study and subdivided into four groups, two in experimental (ovariectomized) and two in control treatment (sham-operated).

**Experimental treatment:** Rats were subjected to surgical removal of both ovaries under general Thiopental anesthesia (40 mg/kg). After Ovariectomy, before closing abdominal walls, penicillin and streptomycin wash was used to reduce inflammation and prevent abscesses formation.

**Control treatment**: Rats were subjected to identical surgical procedure as experimental rats, however without removal of ovaries.

### Experimental design

Two weeks after ovariectomy, animals in both treatments: ovariectomized and sham-operated, were further subdivided into two groups within each treatment. One group in each treatment was receiving 250 mg Maca-GO per kg bw, twice daily (total daily dose of 500 mg Maca-GO per animal) in the form of suspension in distilled water administered to rats by intubation during 28 days of study. The selected dose of tested product was equivalent to 1/20 LD_50_ weight of Maca-GO determined in the acute toxicity assay (22).

Another group in each treatment was given distilled water - identical volume as groups receiving Maca-GO suspension twice daily during the 28 day study.

The following groups were established to observe the effect of Maca-GO on ovariectomized rats and rats with ovaries intact:

- Group KPO - control group of rats with intact-active ovaries receiving distilled water
- Group MPO - group of rats with intact ovaries receiving Maca-GO
- Group KO - control group of ovariectomized rats receiving distilled water
- Group MO - group of ovariectomized rats receiving Maca-GO

All the measurements on four groups of rats involved recording of their responses to individual treatment were established 1 hr after the last treatment dose.

### Procedures and measurements

After four weeks study during which rats in all respective groups were exposed to doses of either Maca-GO or Vehiculum (water only) just before morning intubation (before 9 am), the following pharmacodynamic tests were performed:

- Immobility test (according to Porsolt)
- Sedative (locomotor activity) test
- Anxiolytic (plus-maze) test
- Cognitive activity test

### Immobility test

The test was performed according to Porsolt method (23). Sixty min after administration of Maca-GO or vehiculum, animals were placed in the glass cylinder (18 cm in diameter and 18 cm high) filled with water (25°C). A cylinder was high enough to prevent escape of animal from water. Immobility of rats was measured as time between placing them in water through a swimming interval up to the time point when rat was judged as immobile (remained floating in the water in an upright position, making only occasional movements to keep its head above water level). The total duration of immobility was recorded during maximum 5 min time period, by the observer unaware of which treatment the rats had received.

### Locomotor activity test

Animals were allocated in an actinometer chamber equipped with electromechanical counter - PAN laboratory activity meter (24). Rats’ mobility was recorded as a sum of impulses recorded during 5 minutes of test period. Locomotor activity test was performed on rats one hour after they received an appropriate treatment: Maca-GO or distilled water – vehiculum. Test was conducted in isolation from other animals or people, so as to prevent any adverse effect of disturbance, noise etc.

### Anxiolytic activity

The anxiolytic behavior (was evaluated using the elevated “plus” maze test according to Morris (25) Pellow and File (26) and Fraser *et al*. (27). The test is based on preference of rats to choose darkened areas of maze (closed arms) as oppose to lit areas (150 W lamp placed 1m above the maze) in open maze arms. The rats were placed in the centre of the maze facing one of the open arms. During the 5 min test period the number of the entries into the open and closed arms and the time spent in the open and closed arms was measured automatically by a computer assisted video camera (VideoMot2, TSE, Germany).

### Cognitive activity test

Cognitive activity was measured using passive avoidance test, which allows for assessment of long-term cognitive effects, according to a model presented by Adler *et al*. (28) and Le Merrer and Nogues (29). Animals were placed in a darkened cage (0.425 × 0.404 × 0.456 m) with an attached platform lit with 100 W lamp (28). Principal of the test is based on natural preference of rats to stay in a darkened surrounding. After 2 min adaptation to stay in a dark cage animals were placed on lit platform and measured time for rats returning to the dark cage. With the end of the second test, when rats were in darkened area, an audio signal in conjunction with electric shock was switched on (500 μA) for the 3 sec. duration. After 24 hr and then 48 hr rats were placed again on a lit platform and measured a delay in time for rat returning to the darkened area, taking 180 sec. for maximum extension of time for animal returning to the darkened area. After such time, the observation was terminated. Results were expressed as time factor after 24 hr and 48 hr in relation to the time recorded in the second original attempt and expressed as F_24_ and F_48_ respectively. In the cognitive activity test only those rats were used which in the pre-test (without audio and electric shock stimulation) returned to the darkened area within the 180 sec. from being placed on the lit platform.

### Blood analyses

On the last day of the study, after morning Pharmacodynamic tests, animals were intubated with morning (between 9 am and 10 am) doses of Maca-GO or Vehiculum and one hour later rats were decapitated and blood was collected for blood morphology and for the following biochemical blood analyses: Estrogen (E2), Progesterone (PGS), Cortisol (CT), Adrenocorticotropic Hormone (ACTH), Thyroid Hormones (TSH, T3, T4), Iron (Fe) and lipid profile (Triglycerides, Total Cholesterol, LDL, HDL).

Biochemical assays were conducted by Clinical Diagnostic Laboratory LABO-MED, Poznan, Poland using officially accepted standard clinical procedure on Immulite – DPC equipment. Precision of this technique is monitored by National Center of Quality of Diagnostic Medical Laboratories in Poland and the Laboratory is a participant of the International Quality Control RIQAS maintained by Randox Company.

### Statistical analysis

The data were expressed as means ±SEM and the statistical comparison of results was performed using ANOVA followed by a Duncan post-hoc test.

## RESULTS

### Toxicity (LD_50_) for Maca-GO

From the results obtained in acute toxicity study conducted according to procedure recommended by the AOCD (22) for study chemical substances, it appeared that the highest daily dose of 2 g/kg bw of Maca-GO administered orally by intubation to rats has not visibly affected animals receiving such a high dose of the product and has not resulted in any detectable physiologically-abnormal symptoms. Therefore, a procedure described by Litchfield and Wilcoxon and modified according to Roth (22) has been used to determine LD_50_ dose.

**Figure 1:**
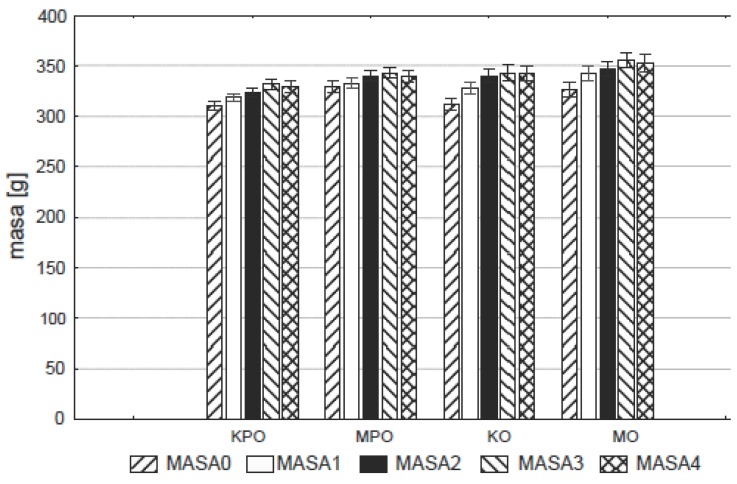
Changes in body weight (masa g) of rats as a result of ingestion of Maca-GO (twice daily dose 250 mg/kg body weight) by ovariectomized (MO) and sham operated (MPO) rats. Results expressed as group mean and standard error of mean (± SEM). KPO, Control-sham operated only (ovaries not removed); KO, Control-operated & ovaries removed; MPO, Maca group-rats sham operated only (ovaries not removed); MO, Maca-rats operated & ovaries removed. MASA0, initial weight; MASA1, body weight after 1 month of study; MASA2, body weight after 2 months of study; MASA3, body weight after 3 months of study; MASA4, body weight after 4 months of study.

All animals survived the LD_50_ toxicity test without any adverse effects noticed in mice or rats on the basis of abnormal behavior, body weight increments (Figure 1) and histopathology of internal organs (liver, spleen, pancreas and testes or ovaries). On the basis of the obtained results summarized in Table 1, the LD_50_ for Maca-GO was >15 g/kg BW as determined on mice and >5 g/kg BW as determined on rats (being the highest doses applied in this study).

**Table 1:** Acute toxicity LD_50_ of Pre-gelatinized Organic Maca (Maca-GO) (Lepidium peruvianum Chacon preparation) determined in a laboratory animal model according to Litchfield and Wilcoxon on male and female, mice and rats

Mice		Rats	
Males	Females	Males	Females

>15	>15	>5	>5

### Biochemistry of Blood

Results of analyses of hormones summarized in Table 2 shows that Maca-GO significantly (*P*<0.05) reduced levels of both E2 and PRG in ovariectomized rats, while in the sham operated animals, E2 serum concentration was reduced with an accompanied increase in PRG (*P*<0.05).

**Table 2:** The effect of four week oral administration of Maca-GO (twice daily dose of 250 mg/kg body weight) on level of hormones in ovariectomized and sham operated rats

GROUP	ESTRADIOL [pg/mL]	Progesteron [ng/mL]	CORTISOL [μg/100 mL]	ACTH [pg/mL]	TSH [μIU/mL]	T3 [pg/mL]	T4 [pg/dL]

KPO	68.8 ± 4.40 (10)^n^	6.52 ± 0.61 (10)	7.03 ± 0.31 (14)	10.61 ± 3.14 (10)	0.18 ± 0.01 (10)^n^	2.38 ± 0.10 (10)	2.62 ± 0.11 (10)
MPO	55.16 ± 3.32+ (10)	8.96 ± 0.45+ (10)	6.69 ± 0.53 (14)	24.55 ± 5.93 (12)	0.14 ± 0.01+ (10)	2.50 ± 0.11 (10)	2.11 ± 0.06+ (14)
KO	77.48 ± 2.83 (12)	2.33 ± 0.19* (12)	5.33 ± 0.43* (12)	47.61 ± 8.48* (12)	0.12 ± 0.01* (12)	3.06 ± 0.11* (16)	1.76 ± 0.08* (16)
MO	59.91 ± 3.87# (14)	1.34 ± 0.13# (14)	3.93 ± 0.35# (14)	7.78 ± 1.86# (14)	0.14 ± 0.01 (14)	2.93 ± 0.09 (14)	1.76 ± 0.05 (18)

KPO, Control-sham operated only (ovaries not removed); KO, Control-operated & ovaries removed; MPO, Maca group-rats sham operated only (ovaries not removed); MO, Maca-rats operated & ovaries removed. Results expressed as group mean and standard error of mean (± SEM).

n number of rats per group. Significance at *P*<0.05;

# MO vs KO;

* KO vs KPO;

+ MPO vs KPO.

Maca-GO significantly (*P*<0.05) reduced level of both glucocorticosteroidal hormones, Cortisol and ACTH in ovariectomized rats, while there were no significant (*P*>0.05) differences recorded in sham operated rats.

Ovariectomy resulted in significant (*P*<0.05) lowering in concentration of TSH but administration of Maca-GO tend to counteract those changes restoring TSH level to concentration observed in sham operated rats with intact ovaries which were receiving Maca-GO. Similar balancing effect of Maca-GO was observed in T3 level which increased in ovariectomized rats, with Maca-GO slightly (*P*>0.05) reducing this tendency. Rats with ovaries intact responded to Maca-Go in an increase of T3 (*P*>0.05) and lowering T4 (*P*<0.05) with ovariectomy lowering the level of T3 further (*P*<0.05) but Maca-Go had no effect in balancing T4 level in ovariectomized rats.

Ovariectomized rats showed significant (*P*<0.05) increase in red blood cells (RBC) count with administration of Maca-GO (Tables 3a and 3b), resulting in significant (*P*<0.05) lowering RBC to the level observed in rats with ovaries intact. This was accompanied by significant (*P*<0.05) reduction in hemoglobin and hematocrit levels, while concentration of both hemoglobin in red cells and total serum Fe significantly increased as a result of Maca-Go intake by ovariectomized rats only, while in rats with intact ovaries, contents of serum Fe was significantly (*P*<0.05) reduced. All levels recorded in blood morphology were within ranges considered as physiologically-normal for rats according to Altman & Dittmer (30) and Saitoch *et al*. (31).

**Table 3a:** The effect of administration of Maca-GO (twice daily 250 mg/kg live weight) on blood morphology in ovariectomized and sham operated rats

GROUP	RBC [M/μL]	WBC [K/μL]	LYM [K/μL]	MID [K/μL]	GRAN [K/μL]	PLT [K/μL]

KPO	7.30 ± 0.10 (11)^n^	7.15 ± 0.5 1(11)	5.13 ± 0.37 (11)	0.95 ± 0.07 (11)	1.03 ± 0.07 (11)	850.36 ± 41.28 (11)
MPO	7.20 ± 0.08 (13)	6.01 ± 0.29 (12)	4.43 ± 0.21 (12)	0.81 ± 0.04 (12)	0.71 ± 0.08+ (12)	862.33 ± 23.26 (12)
KO	7.83 ± 0.10* (14)	6.99 ± 0.38 (14)	5.32 ± 0.30 (14)	0.88 ± 0.04 (14)	0.83 ± 0.06 (12)	803.93 ± 29.09 (14)
MO	7.26 ± 0.10# (18)	7.43 ± 0.48 (15)	5.69 ± 0.38 (16)	0.88 ± 0.07 (16)	0.68 ± 0.07 (14)	795.13 ± 49.46 (16)

KPO, Control-sham operated only (ovaries not removed); KO, Control-operated & ovaries removed; MPO, Maca group-rats sham operated only (ovaries not removed); MO, Maca- rats operated & ovaries removed; RBC, Red blood cells; WBC, White blood cells; LYM, Lymphocytes; MID, Monocytes; GRAN, Granulocytes; PLT, Platelets. Results expressed as group mean and standard error of mean (± SEM).

n number of rats per group. Normal levels for rats (30, 31): RBC [M/μL]=7.2-9.6; WBC [K/μL]=5.0-25.0; LYM [K/μL]=4.5-16; MID [K/μL]=0-1.3; GRAN [K/μL]=93-133; PLT [K/μL]=751-1167. Significance at *P*<0.05;

# MO vs KO;

* KO vs KPO;

+ MPO vs KPO.

**Table 3b:** The effect of administration of Maca-GO (twice daily 250 mg/kg body weight) on blood morphology in ovariectomized and sham operated rats

GROUP	HGB [g/100 ml]	HCT [%]	MCV [mcl]	MCH [pg]	MCHC [g/dL]	RDW [%]	Fe [μg/mL]

KPO	14.54 ± 0.19 (13)	36.66 ± 0.61 (13)	49.92 ± 0.45 (13)	19.90 ± 0.18 (13)	39.81 ± 0.19 (13)	14.69 ± 0.27 (13)	281.00 ± 4.07 (10)
MPO	14.33 ± 0.14 (12)	36.45 ± 0.43 (12)	50.93 ± 0.38 (14)	20.04 ± 0.12 (14)	39.36 ± 0.25 (14)	14.38 ± 0.27 (12)	257.50 ± 4.59+ (12)
KO	14.62 ± 0.14 (16)	38.76 ± 0.59* (16)	48.94 ± 0.77 (16)	18.82 ± 0.14* (14)	37.91 ± 0.32* (16)	14.69 ± 0.24 (14)	189.25 ± 4.11* (16)
MO	14.08 ± 0.19# (18)	35.82 ± 0.55# (18)	49.47 ± 0.47 (17)	19.44 ± 0.17# (16)	39.47 ± 0.21# (18)	15.15 ± 0.34 (16)	193.57 ± 1.63 (14)

KPO, Control-sham operated only (ovaries not removed); KO, Control-operated & ovaries removed; MPO, Maca group-rats sham operated only (ovaries not removed); MO, Maca-rats operated & ovaries removed; HGB, hemoglobin; HCT, hematocrit; MCV, volume of erythrocytes; MCH, weight of hemoglobin in erythrocytes; MCHC, concentration of hemoglobin in red cells; RDW, deviation in size of erythrocytes. Fe, Total Iron. Results expressed as group mean and standard error of mean (± SEM). Normal levels for rats (30, 31): HGB [g/dL]=12.0-17.5; HCT [%]=39-53; MCV [fL]=49-61; MCH [pg]=16.4-20.0; MCHC [g/dL]=32.1-34.9. Significance at *P*<0.05;

# MO vs KO;

* KO vs KPO;

+ MPO vs KPO.

After Ovariectomy, Maca-GO have significantly (*P*<0.05) increased serum Total Cholesterol, HDL and LDL (Table 4), but has no significant effect (*P*>0.05) on Triglycerides, Cholesterol and HDL, with an observed trend to slight reduction of triglycerides, Cholesterol and HDL in both, ovariectomized and non-ovariectomized rats after exposure to Maca-GO.

**Table 4:** Analysis of lipid fractions in blood of ovariectomized and sham operated rats after administration of Maca-GO (twice daily 250 mg/kg body weight)

GROUP	CHOLESTEROL [mg/100 ml]	HDL [mg/100 ml]	LDL [mg/10 ml]	TGD [mg/100 ml]

KPO	63.9 ± 1.5 (14)^n^	33.6 ± 0.9 (14)	5.7 ± 0.2 (14)	105.8 ± 5.1 (10)
MPO	61.3 ± 1.9 (14)	32.1 ± 0.7 (14)	5.4 ± 0.3 (14)	110.6 ± 12.2 (10)
KO	72.6 ± 1.8* (16)	36.8 ± 0.6* (16)	6.1 ± 0.2 (16)	98.0 ± 3.7 (12)
MO	68.8 ± 1.7 (18)	35.2 ± 0.7 (18)	6.2 ± 0.2 (18)	90.0 ± 4.9 (14)

KPO, Control-sham operated only (ovaries not removed); KO, Control-operated & ovaries removed; MPO, Maca group-rats sham operated only (ovaries not removed); MO, Maca-rats operated & ovaries removed. Results expressed as group mean and standard error of mean (± SEM).

n number of rats per group. Normal levels for rats: Cholesterol = 40-130 mg/100ml. Significance at *P*<0.05.

* KO vs KPO.

### Pharmacodynamic study

**Figure 2:**
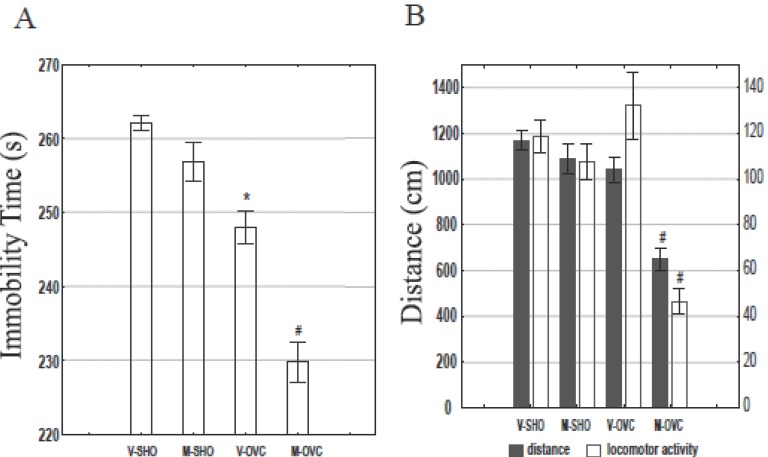
The effect of Maca-GO treatment (twice daily 250 mg/kg body weight) on anti-depressive activity in Porsolt immobility test (A) and sedative - anxiolytic and locomotor activity test (B) in ovariectomized and sham operated rats. Results expressed as group mean and standard error of mean (± SEM). V, vehicle (water); M, Maca-GO; SHO, sham-operated group; OVC, ovariectomized group. Significant differences at *P*<0.05: *vs V-SHO; #vs V-OVC.

**Immobility test (according to Porsolt).** Ovariectomy resulted in significant (*P*<0.05) shortening of time during which rats stayed immobile (Figure 2A). Maca-GO administration to ovariectomized rats led to significant (*P*<0.05) reduction in their immobility time in Porsolt (24) forced swimming test, while there was only slight, statistically non-significant (*P*>0.05) effect of Maca-GO administration on rats with intact ovaries.

**Sedative (locomotor activity) test.** Sedative effect of Maca-GO on ovariectomized and sham operated rats was assessed by measuring distance (cm) covered by rats and frequency of their movements during 5 min test period (Figure 2B). Maca-GO administered to ovariectomized rats resulted in a significant (*P*<0.05) sedative effect expressed in lower mobility (number of impulses = movements) and lesser distance (cm) covered by rats during the 5 min test. There was no such effect of Maca-GO observed in non-ovariectomized rats, which showed higher motoric activity in both measurements as compared to ovariectomized rats.

**Anxiolytic (plus-maze) test.** Similar results were obtained in an anxiolytic test with the use of a “plus-maze test” in which ovariectomized rats significantly (*P*<0.05) reduced their entries - visits into lit arms of maze and significantly (*P*<0.05) extended time of staying in darkened area of maze (Table 5). Ovariectomized rats which were administered Maca-GO had less entries into lit area of maze with more frequent and longer stay in darkened area which may be linked to sedative effect of Maca-GO on ovariectomized rats, with no such effect being observed in non-ovariectomized rats.

**Table 5:** The effect of administration of Maca-GO (twice daily 250 mg/kg body weight) on results obtained in an anxiolytic test behavior of ovariectomized and sham operated rats placed in an elevated-plus maze

GROUP	“Cross” Test				
	No entries into darkened area	No entries in a lit area	Distance[cm]	Time spent in darkened area [sec.]	Time spent in a lit area [sec.]

KPO	7 ± 1 (13)^n^	6 ± 1 (13)	1167.9 ± 44.9 (11)	52.1 ± 4.9 (11)	20.0 ± 3.9 (11)
MPO	7 ± 1 (12)	6 ± 1 (12)	1089.8 ± 68.3 (10)	45.7 ± 5.3 (10)	18.9 ± 5.1 (10)
KO	7 ± 1 (14)	9 ± 2 (14)	1040.4 ± 56.5 (12)	43.9 ± 5.4 (12)	23.1 ± 2.1 (12)
MO	8 ± 1 (14)	3 ± 0.3# (18)	649.0 ± 53.6# (14)	72.2 ± 14.4# (14)	14.8 ± 3.1 (14)

KPO, Control-sham operated only (ovaries not removed); KO, Control-operated & ovaries removed; MPO, Maca group-rats sham operated only (ovaries not removed); MO, Maca-rats operated & ovaries removed. Results expressed as group mean and standard error of mean (± SEM).

n number of rats per group. Significance at *P*<0.05;

# MO vs KO.

**Figure 3:**
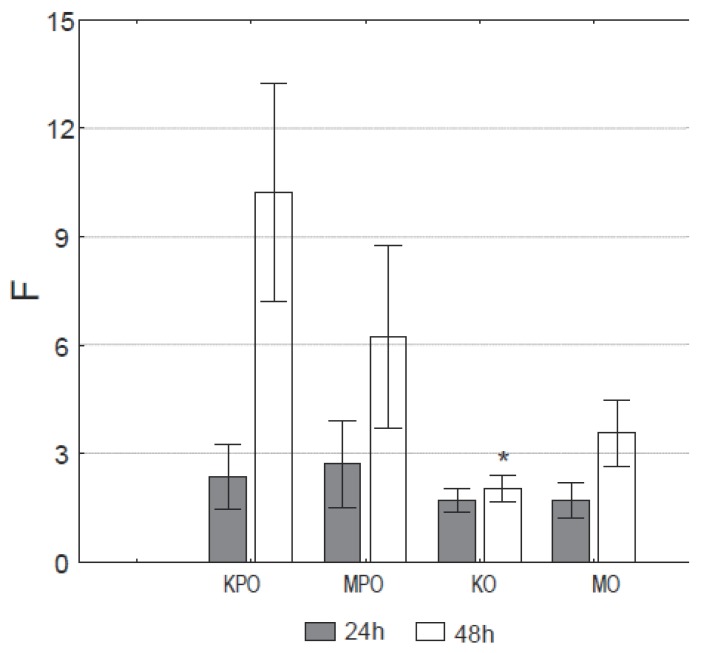
The effect of administration of Maca-GO (twice daily. 250 mg/kg body weight) on long-term memory retention in rats expressed as Memory Index: F24 and F48 indicating time needed for rats to find their way back to the initial “reward point” after 24 hr and 48 hr from the training session respectively. Results expressed as group mean and standard error of mean (± SEM). KPO, Control-sham operated only (ovaries not removed); KO, Control-operated & ovaries removed; MPO, Maca group-rats sham operated only (ovaries not removed); MO, Maca-rats operated & ovaries removed.

**Cognitive Test.** Ovariectomized rats showed significant (*P*<0.05) reduction in cognitive functions as measured by passive avoidance test expressed in the form of Cognitive Index F_24_ and F_48_ (Figure 3). Statistically significant (*P*<0.05) difference was recorded in F_48_ showing a decline in long-term (after 48hr) cognitive functions in ovariectomized rats, as compared to rats with intact ovaries. Administration of Maca-GO to ovariectomized rats has slightly, although statistically not significantly (*P*>0.05), improved long-term cognitive functions (F_48_) but non-ovariectomized rats showed opposite, also non-significant (*P*>0.05) effect on the same measurement.

## DISCUSSIONS

On the basis of the obtained results, adopting recommendations by Hodge and Sterner as per the OECD (22), the LD_50_ for Maca-GO for oral applications was established as 15 g/kg body weight – a way above the OECD 2 g/kg limit. According to obtained results it is reasonable to suppose that Maca-GO doesn’t present health hazard for humans consuming this product at the dose up to 15 g/kg (which is the equivalent to approximately 1 kg Maca-GO for 66 kg body weight man/women or nearly 1.3 kg for 85 kg person).

The results obtained in this study confirm previously obtained observations on rats (12) exposed to various levels of Maca-GO (0.75 g/kg and 7.5 g/kg body weight of rats) where no detectable negative physiological, clinical, history-pathological nor toxic effects existed which could be attributed to the used doses of Maca-GO and administered to rats during either 28 days or 90 days experimental periods.

From reported in the literature (13, 22) long list of physiological and physical benefits of Maca traditionally used for centuries as vegetable with “medicinal properties” by natives of Peru and now gaining popularity as a dietary supplement in the USA and Europe, the followings appear to be of importance for menopausal women: balancing hormonal secretion, stimulation of body metabolism, increase in energy and vitality (32) stress reduction, antidepressant activity, memory improvement, enhancement of sexual drive (7, 21, 33). Since hormones affect entire spectrum of metabolic and psychological responses of women during pre-and postmenopausal stages, in an adopted laboratory animal model, both ovariectomized rats - in their, surgically-induced physiological status resembling post-menopause and non-ovariectomized, sexually experienced rats with active ovaries - as pre-menopausal animals were used. Analyzing biochemical blood indices and results from pharmacodynamic tests, an attempt has been made to identify metabolic axis, along which Maca-GO as a dietary supplement, may exhibit its action on post- and pre-menopausal rats.

Therapeutic properties of Maca roots are linked to alkaloids identified in 1960s by Chacon (1) and later confirmed by other researchers, however in recent years other groups of active constituents were reported such as polyunsaturated acids and their amides (“macaine” and “macamide”), sterols (campesterol, stigmasteroland beta-sitosterol, and aromatic glucosinolates (benzyl and p-methoxybenzyl glucosinolates and their derived isothiocyanates) (9, 13-16). There is no single “active” component identified which would be agreed to as a “functional” or “active” marker for Maca, therefore in this and follow-up study, Maca in its entirety - as traditionally Maca has been referred to, is being used after an application of a standard pre-gelatinization process and referred to as pre-gelatinized organic Maca - Maca-GO.

Previously published results from preliminary study (11) demonstrated that Maca-GO used for extended period of time (8 months) in postmenopausal women significantly increased progesterone (PRG) and only slightly reduced E2 level. A shorter time of Maca-GO administration (2 months) significantly reduced PRG in relation to placebo level while the E2 was not affected. In this study, Maca-GO significantly reduced both E2 and PRG in “postmenopausal” rats, while there was a significant decrease in E2 at significantly increased PRG in “pre-menopausal” rats. This effect of Maca-Go on rats with active ovaries may be extremely valuable when extrapolating results from the laboratory model to potential responses in perimenopausal women, who are subjected to fluctuations in E2/progesterone ratio resulting in relative increase in E2 level due to progressive decline in secretion of PRG by *corpus luteum* (21). The observed in this study significant reduction in both E2 and Progesterone after administration of Maca-GO to ovariectomized rats needs to be interpreted with caution since it is reasonable to suppose that such effect in women with inactive ovaries would not be physiologically-beneficial.

On the other hand, observed in this study antidepressive effect of Maca-GO in ovariectomized rats may add to difficulty in interpretation of trends in changes of the two hormones, particularly in view of lack in relationship between antidepressive effect and lowered E2 level, since reduced level of E2 has been shown in clinical study on laboratory animals as responsible for development of the depressive symptoms (34). Also, observed reduction in both ACTH and Cortisol levels which was accompanied by the demonstrated antidepressive effect of Maca-GO on ovariectomized rats is similar to reported by DeMoranville and Jackson (35) and Sapolsky (36), who, in people suffering from illnesses and in clinical models to study depression, observed an increase in Cortisol with progression of depressive state. From this point of view, Maca-GO appears to have positive effect on alleviation of depression with a simultaneous sedative effect as well. This could be ascribed to high daily dose of Maca-GO applied to rats in this study, since doses of 15 and 7.5 mg/kg used over 15 days, resulted in a stimulation of motoric function in male rats (37). There maybe a difference in motoric responses to Maca-Go by male and female rats, but this aspect has not been reported yet in literature available to authors’ to-date.

Observed in this study improved long-term cognitive ability in ovariectomized rats receiving Maca-GO is worthy to be emphasized, since changes in mood and disturbance of cognitive functions, which are frequently considered as first signs of ovarian dysfunction in menopausal women, may be alleviated, reduced or delayed by use of Maca-GO prior-to, during transition and at postmenopausal stage. This may be further supported by the fact of very low toxic status of Maca-GO, with its LD_50_ determined at >15 g/kg body weight, hence, safe for oral use and being well tolerated as dietary supplement (equivalent to approximately 1 kg oral intake of Maca-GO per day by women of 66 kg BW). Supporting this assumption is the dose of 500 mg/kg BW per day used in this four week long study (1/20 of LD_50_), which has not affected morphologic blood parameters, remaining within the ranges considered as normal for rats (30, 38). Long-term administration of Maca-Go to ovariectomized rats resulted in slight reduction of blood cholesterol and triglycerides which may indicate its positive effect on metabolism of lipids, also observed by other authors (39).

Results obtained in this model laboratory study on Maca-GO are encouraging enough to justify setting up further laboratory and/or clinical study on postmenopausal women to validate positive effects of this preparation as a dietary supplement on human subjects under average everyday situation. In this study, Maca-GO was administered at one, arbitrary-chosen level only, which eliminated detection of possible variation in daily dose-related individual responses to different level of intake, as well as due to potential different sensitivity of rats to Maca, which was observed by Muller (6) in her clinical practice on women. This would need to be studied further. Also, it will be essential to compare observed in this study antidepressive and sedative effect of Maca-GO, with known antidepressants such as fluoxetine (40), which is recommended in treating menopausal women for depression symptoms (41, 42). Fluoxetine, through inhibition of reverse uptake of serotonin, is believed to mimic action of estrogen (43), responsible for elevation in level of serotonin, an important mood elevating neurotransmitter in the brain.

Interpreting behavior of rats in pharmacodynamic model as applied in this study, Maca-GO possesses typical antidepressant-like profile when administered to ovariectomized rats. This anti-depressive effect was associated with lowering of Cortisol and ACTH levels which may indicate a sedative effect of Maca-GO on ovariectomized rats. In study reported by Lowicka *et al*. (44), where the effect of administering gelatinized Maca to ovariectomized rats was compared with fluoxetine - a known antidepressant (40), the observed effects of Maca on spontaneous activity and antidepressive-like activity were different from those induced by fluoxetine. Therefore, it may be assumed that mechanisms other than serotoninergic system are involved in pharmacological action of Maca, which also, differently affected ovariectomized and non-ovariectomized rats. Previously reported results obtained on laboratory animals with intact ovaries (12) showed similar effect as observed in this study on both ovariectomized and non-ovariectomized rats, that Maca-GO have positive effect on both, reduction in blood cortisol and therefore, lowering susceptibility of rats to stress factors and its sedative effect on laboratory animals, the properties also reported by Lopez Fondo *et al*. (45).

Observed in this study, a decrease in E2 and an increase in E2-to-PRG ratio in ovariectomized rats, as a result of Maca-GO ingestion (ratio from 33 to 44), contradict recorded antidepressive effect of this preparation in a laboratory model on rats. However, in non-ovariectomized rats, the same ratio was reduced (from 10 to 6), which may explain slight positive antidepressive effect of Maca-Go and support observations reported in the literature (43, 46). Also Lucille (47) emphasized that the balance between progesterone, estradiol and thyroid function is one of the key factors in female maintaining the hormonal balance during the reproduction years and in menopause. It is a key function of progesterone to control estradiol and prevent negative effects of its dominance as well as to support thyroid function in maintaining growth, healthy bone metabolism and balancing psychological equilibrium in female organism during their reproductive and then in a menopausal stage. In this study such a relationship has been confirmed in non-ovariectomized rats in relation to E2 and PRG only, but has not been confirmed in ovariectomized animals, since in those animals Maca-GO administration resulted in both estradiol and PRG reduction with no significant effect on thyroid profiles.

Maca-GO reduced serum Iron level in non-ovariectomized rats and had no effect on ovariectomized animals which does not support observations obtained in earlier laboratory trial on male and female rats (12) nor confirmed the results reported in the literature (38), suggesting that Maca may stimulate the absorption of dietary Iron from the digestive tract.

In the next part of this paper (Part II), blood biochemistry and pharmacodynamics in a laboratory model on rats as reported here, are follow-up by clinical study on early-postmenopausal women volunteers, who self-administered Maca-GO in various length and time intervals intermittently with placebo in a double blind, coordinated multi-centre study in which similar biochemical measurements and testing of physical and psychological menopausal symptoms were conducted in “human clinical model”.

## CONCLUSIONS

- Pre-Gelatinized Maca Organic - *Lepidium peruvianum* Chacon (Maca-GO) was characterized by low toxicity: LD_50_ > 15.0 g/kg BW for mice and LD_50_ >5 g/kg BW for rats which is classified as fifth class of toxicity – substance practically non-toxic.
- Maca-GO administered twice daily 250 mg/kg BW during four weeks study, to sham operated rats with functioning ovaries, lowered blood E2 and increased PRG levels.
- After Ovariectomy, Maca-GO resulted in lowering both E2 and PRG.
- Ovariectomized animals showed higher concentrations of cortisol and ACTH as compared to non-ovariectomized rats.
- Maca-GO led to lowering Cortisol and ACTH levels in the ovariectomized rats, while there was no difference in response to Maca-GO in sham operated animals.
- Maca-GO restored changes in ACTH which occurred after ovariectomy, however did not normalized Cortisol level.
- After 4 weeks administration of Maca-GO, there was a slight reduction in blood cholesterol and triglycerides.
- Although Maca-GO changed some blood morphology parameters in rats such as hemoglobin or granulocytes, all measured parameters were maintained within the range considered for rats as normal.
- Results of pharmacodynamic tests suggest that Maca-GO exhibited typical antidepressant-like and sedative, but not anxiolytic effect on ovariectomized rats, which was associated with lowering in serum cortisol and ACTH levels.
- Rats in ovariectomized group receiving Maca-GO, showed impairment of spontaneous activity and number of entries into the dark arm, however no such results were recorded in non- ovariectomized rats.
- Maca-GO improved long-term cognitive functions (F_48_) in ovariectomized rats.
- On the basis of observations made on sexually-experienced rats with functional ovaries, it is reasonable to suggest that Maca-GO may be also of value in the treatment of some depressive symptoms during perimenopause period.
